# Ultrasonographic Versus Anthropometric Measurement of Subarachnoid Space Depth in Adults Undergoing Spinal Anaesthesia: A Prospective Comparative Study

**DOI:** 10.7759/cureus.92885

**Published:** 2025-09-21

**Authors:** Dharani M, Geetha Soundarya Udayakumar, Yogarajan Ramalingam

**Affiliations:** 1 Anaesthesiology, Sree Balaji Medical College and Hospital, Chennai, IND

**Keywords:** anthropometric measurements, obesity, spinal anaesthesia, subarachnoid space depth, ultrasound guided subarachnoid space depth

## Abstract

Background: Accurate identification of subarachnoid space depth (SSD) is crucial for successful spinal anesthesia. While traditional anthropometric methods are commonly used, their reliability varies, particularly in obese patients and those with spinal deformities. Ultrasonography offers real-time visualization but requires validation against established techniques.

Methods: A prospective observational study was conducted on 56 adults (18-65 years) scheduled for elective surgeries under spinal anesthesia. Ultrasonographic SSD (USG-SSD) was measured preoperatively at the L3-L4 level using a curvilinear probe, while anthropometric SSD (ANTHRO-SSD) was calculated using Bonadio’s formula. The actual SSD was recorded during needle insertion. Statistical analysis included Bland-Altman agreement, Pearson correlation, and receiver-operating characteristic (ROC) curves.

Results: USG-SSD (4.5 ± 0.6 cm) closely matched actual SSD (4.6 ± 0.5 cm; with p value = 0.12), whereas ANTHRO-SSD overestimated depth (5.1 ± 0.7 cm; p< 0.001). Ultrasonography showed superior agreement (limits: −0.6 to +0.4 cm), lower errors (MAE: 0.3 cm vs. 0.7 cm), and stronger correlation (r = 0.92 vs. 0.75). Accuracy was consistent across BMI categories, unlike anthropometry, which overestimated SSD in obese patients (error: 1.1 ± 0.5 cm). Ultrasonography achieved 87.5% first-attempt success and discriminated difficult cases (SSD >5 cm) effectively (area under the ROC curve (AUC): 0.93 vs. 0.69 for anthropometry).

Conclusion: Ultrasound-guided SSD estimation is more accurate and reliable than anthropometric methods, particularly in high-BMI populations. Its integration may improve procedural success rates and reduce complications.

## Introduction

Spinal anaesthesia is a widely used regional anaesthetic technique for surgeries involving the lower abdomen, pelvis, and lower extremities [1]. Its success relies on accurate identification of the subarachnoid space (SAS) for cerebrospinal fluid (CSF) aspiration and drug administration [2]. Traditionally, this has been achieved using surface anatomical landmarks, but this method is prone to errors, particularly in obese patients, those with spinal deformities, or edema [3]. Ultrasonography has emerged as a valuable tool, offering real-time visualization of spinal structures and improving needle placement precision [4]. Anthropometric measurements such as BMI, body surface area (BSA), and waist circumference have also been used to predict SAS depth, but their reliability varies across different populations [5].

Spinal anaesthesia was first introduced by August Bier in 1898, revolutionizing surgical pain management [5]. The technique traditionally relies on palpation of anatomical landmarks such as Tuffier's line (intercristal line) to identify the lumbar SAS [6]. However, landmark-based techniques are often unreliable in patients with difficult anatomy, such as obesity or spinal deformities [7]. Studies have shown that these methods can lead to multiple needle attempts, increasing the risk of complications such as post-dural puncture headache, nerve injury, and infection [8].

Ultrasound-guided neuraxial anaesthesia has gained prominence due to its ability to visualize deep anatomical structures [9]. Research demonstrates that real-time ultrasound guidance significantly improves first-attempt success rates, reduces procedural time, and enhances patient comfort [10]. Pre-procedural ultrasound measurement of the skin-to-SAS distance allows for individualized depth estimation, which is particularly beneficial in patients where anthropometric predictions may be unreliable, such as those with obesity or spinal abnormalities [11].

Several formulas have been proposed to estimate SAS depth using anthropometric data. Chong et al. derived a pediatric-specific model [12]. Bonadio's formula, which incorporates BSA, is another commonly used predictor [13]. However, these formulas may not be universally applicable due to variations in body habitus and spinal anatomy across different ethnic groups. Some studies report strong correlations between BMI and SAS depth, while others find no significant association with factors like age or gender [14,15].

The primary rationale for this study is to determine whether ultrasound-guided measurement of SAS depth is more accurate than anthropometric predictions. Improved accuracy could lead to higher procedural success rates, fewer complications, and better patient outcomes. Additionally, pre-procedural ultrasound assessment allows for personalized anaesthesia, optimizing drug delivery based on individual anatomical variations. The study aims to assess the association between ultrasonographic measurement, anthropometric measurement, and actual depth of SAS in all adult patients undergoing spinal anaesthesia during the study period. The objective is to compare and contrast the ultrasonographic measurement of SSD with actual needle insertion versus anthropometric measurements, thereby proving that the use of ultrasound for estimation of SSD is more accurate than anthropometric measurements.

## Materials and methods

This prospective observational study was conducted in the Department of Anaesthesia at Sree Balaji Medical College & Hospital, Chennai, India, from November 2023 to November 2024, involving adult patients aged 18-65 years who were scheduled for elective surgeries under spinal anesthesia and provided informed consent. The study protocol was approved by the Institutional Research and Ethics Committee, Sree Balaji Medical College & Hospital (Reference number: 002/SBMCH/IHEC/2023/2044). The study was registered at the Clinical Trial Registry-India (registration number: CTRI/2024/10/076045).

Eligibility criteria

Inclusion Criteria

Inclusion criteria were: Both male and female patients with American Society of Anesthesiologists (ASA) physical status I and II, aged 18-65 years, who underwent various elective surgeries under spinal anesthesia.

Exclusion Criteria

The following patients were excluded: (i) Patients with local pathology at the site of injection, (ii) Patients belonging to ASA grade III or above, (iii) Pregnant patients, (iv) Patients with bleeding disorders, (v) Patients unable to maintain a sitting position without assistance, (vi) Patients with kyphosis, lordosis, scoliosis, or failed spinal anesthesia block (SAB), (vii) Patients who did not cooperate or refused to participate, and (viii) Patients scheduled for emergency surgery.

Sample size calculation

Based on the study conducted by Tyagi et al. [16], the sample size was calculated based on the correlation between ultrasound-guided SAS depth, anthropometric-based SAS depth, and actual depth, with 80% power, an alpha error of 5%, assuming a population coefficient of 0.5%. The total sample size was calculated as 56 patients. 

Data collection

The data collection process was conducted by trained researchers and anesthesiologists using standardized tools and protocols. A semi-structured questionnaire served as the primary instrument for gathering comprehensive demographic information, including age, gender, BMI, waist circumference, and arm circumference. Additional relevant clinical parameters were systematically recorded in a predesigned proforma to ensure consistency and completeness of data collection across all study participants. 

Preoperative anthropometric measurements, including height (using a fixed wall tape) and weight (using a standard weighing machine), were taken, and BSA was calculated using the Mosteller formula:

$\text{BSA} \,(m^{2}) = \sqrt{\frac{\text{height (cm)} \times \text{weight (kg)}}{3600}}$

Subarachnoid space depth (SSD) was calculated using Bonadio’s formula:

SSD (cm) = 0.77 cm + 2.56 × BSA (m²)

Preoperative ultrasound assessment of the lumbar spine was performed in the sitting position, using a low-frequency curvilinear probe. L3-L4 space was identified and marked, and the distance from the skin to the posterior complex was measured using built-in calipers. For the administration of spinal anesthesia, patients were positioned sitting with their backs flexed. A 25-gauge Quincke spinal needle (3.5 inches/8.9 cm) was inserted at the L3-L4 space using a midline approach under strict aseptic technique. Once CSF flow was confirmed, the needle was marked at the skin level with a sterile marker. The needle was then withdrawn, and the actual insertion depth was measured with a standard scale for comparison with the predicted values.

Data analysis 

Statistical analysis was performed using IBM SPSS Statistics for Windows, version 26.0 (IBM Corp., Armonk, New York, United States) by presenting categorical variables as numbers and percentages (%), while continuous variables were expressed as mean ± standard deviation (SD) along with median values. Data normality was assessed using the Kolmogorov-Smirnov test, with non-parametric tests employed when normality assumptions were violated. Quantitative variables were compared using Independent t-tests or Mann-Whitney tests (for non-normal distributions), while qualitative variables were analyzed through Chi-square or Fisher's exact tests, with a p-value < 0.05 considered statistically significant. 

## Results

A total of 56 patients meeting the inclusion criteria were enrolled in the study. The demographic and clinical characteristics are summarized in Table 1. The mean age was 45.2 ± 12.3 years, with a balanced gender distribution. The mean BMI was 28.4 ± 3.8 kg/m², indicating a predominantly overweight population. Most patients were ASA I (62.5%), with the remaining classified as ASA II (37.5%).

**Table 1:** Demographic and clinical characteristics of study participants (N=56) BMI: body mass index; ASA: American Society of Anaesthesiology; SD: standard deviation

Variable	Value
Age (years), mean ± SD	45.2 ± 12.3
Gender, n (%)	
- Male	32 (57.1%)
- Female	24 (42.9%)
BMI (kg/m²), mean ± SD	28.4 ± 3.8
ASA Physical Status, n (%)	
- ASA I	35 (62.5%)
- ASA II	21 (37.5%)
Waist Circumference (cm), mean ± SD	88.5 ± 9.2
Arm Circumference (cm), mean ± SD	28.3 ± 3.5

**Figure 1:**
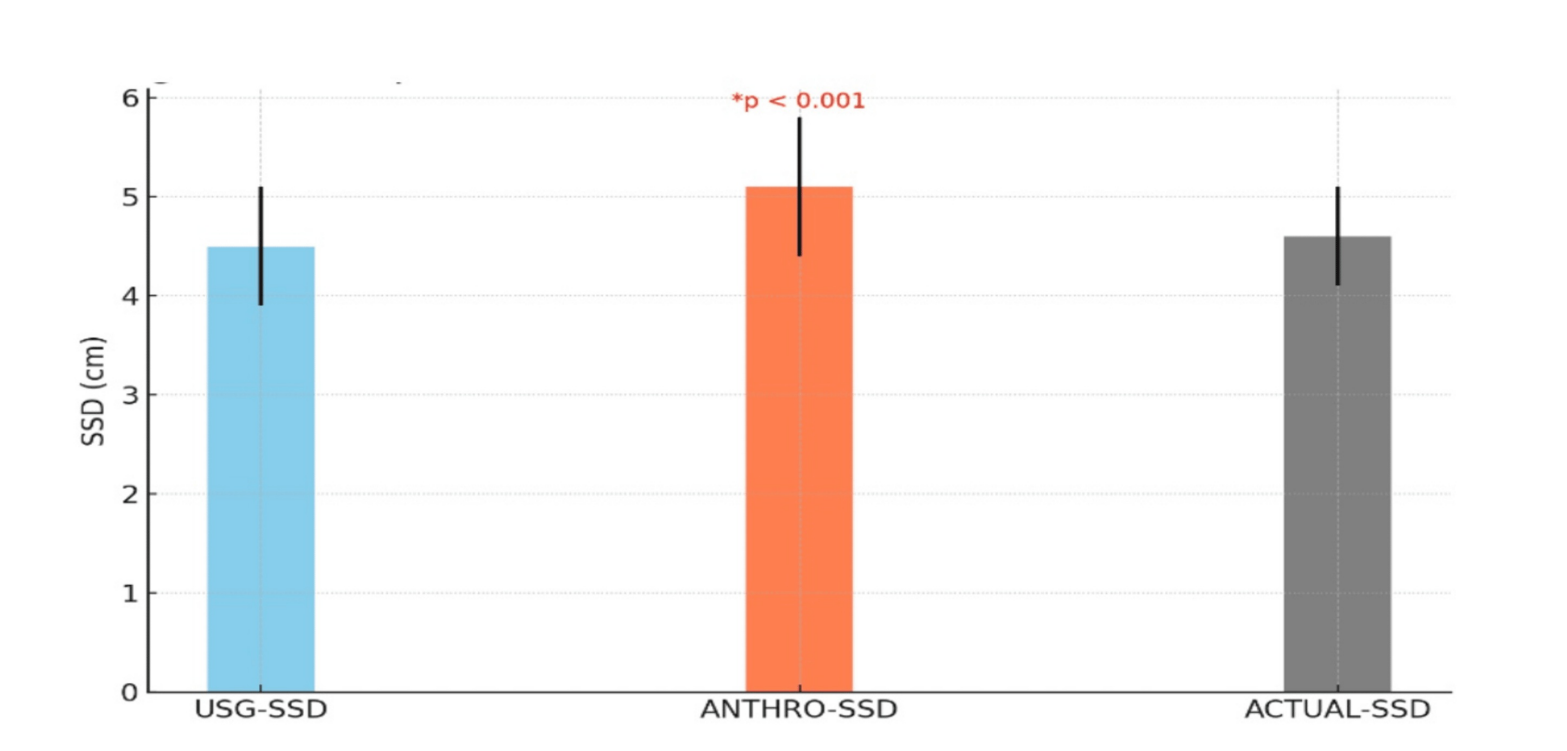
Comparison of subarachnoid space depth measurements (cm) USG-SSD: ultrasound-measured subarachnoid space depth; ANTHRO-SSD: Anthropometric-measured subarachnoid space depth; ACTUAL-SSD: actual subarachnoid space depth

The ultrasound-measured SSD (USG-SSD), anthropometrically estimated SSD (ANTHRO-SSD), and actual needle-measured SSD were compared. USG-SSD (4.5 ± 0.6 cm) showed no significant difference from the actual SSD (p=0.12), while ANTHRO-SSD (5.1 ± 0.7 cm) overestimated the depth compared to the actual SSD (p<0.001), as shown in Figure 1.

**Figure 2:**
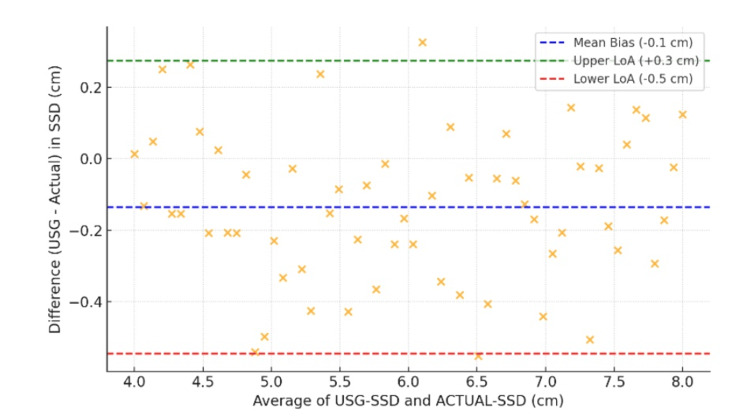
Bland-Altman Analysis (USG-SSD vs. ACTUAL-SSD) USG-SSD: ultrasound-measured subarachnoid space depth; ACTUAL-SSD: actual subarachnoid space depth

The mean bias was -0.1 cm, indicating that ultrasound slightly underestimated the depth, with limits of agreement (LoA) ranging from -0.6 cm to +0.4 cm (95% CI). Figure 2 illustrates that 94.6% of measurements fell within LoA, indicating good agreement.

**Figure 3:**
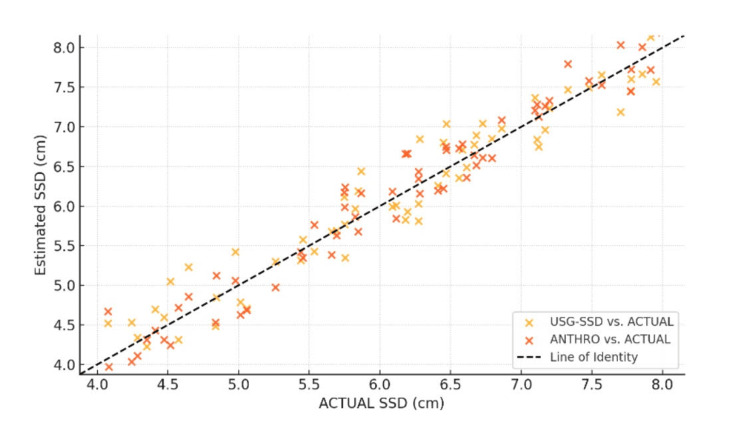
Pearson correlation analysis USG-SSD: ultrasound-measured subarachnoid space depth; ANTHRO-SSD: anthropometric-measured subarachnoid space depth; ACTUAL-SSD: actual subarachnoid space depth

Ultrasound showed a stronger correlation (r=0.92) with actual SSD than anthropometry (r=0.75), with a significant p-value of <0.001 as shown in Figure 3.

USG-SSD had lower errors (mean absolute error (MAE)=0.3 cm) compared to ANTHRO-SSD (MAE=0.7 cm). Root mean square error (RMSE) was significantly lower for ultrasound, indicating higher precision, as shown in Table 2.

**Table 2:** Prediction errors for ultrasonographic and anthropometric methods USG-SSD: ultrasound-measured subarachnoid space depth; ANTHRO-SSD: Anthropometric-measured subarachnoid space depth

Method	Mean Absolute Error (MAE, cm)	Root Mean Square Error (RMSE, cm)
USG-SSD	0.3	0.4
ANTHRO-SSD	0.7	0.9

Ultrasound remained more accurate across all BMI groups, with anthropometry showing increasing error for obese patients, as shown in Table 3.

**Table 3:** Comparison of subarachnoid space depth accuracy by BMI category BMI: body mass index; USG-SSD: ultrasound-measured subarachnoid space depth; ANTHRO-SSD: Anthropometric-measured subarachnoid space depth

BMI Category (kh/m^2^)	USG-SSD Error (cm)	ANTHRO-SSD Error (cm)	p-value
Normal (18.5–24.9)	0.2 ± 0.1	0.5 ± 0.3	0.003
Overweight (25–29.9)	0.3 ± 0.2	0.8 ± 0.4	<0.001
Obese (≥30)	0.4 ± 0.2	1.1 ± 0.5	<0.001

**Figure 4:**
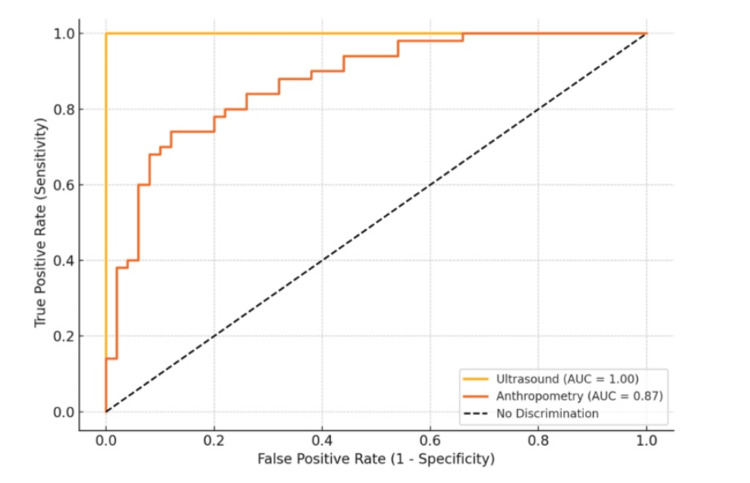
Receiver-operating characteristic (ROC) curve analysis AUC: area under the ROC curve

Success rate during the first attempt was 87.5% (49/56), whereas the second attempt success rate was 7.1% (4/56). The use of ultrasound increased the success rate on the first attempt and reduced complications, with only one case (1.8%) of post-dural puncture headache (PDPH) and three cases (5.4%) requiring multiple attempts (>2). ROC curves confirmed ultrasonography's excellent discrimination (AUC>0.9), as shown in Figure 4.

## Discussion

This prospective observational study provides compelling evidence supporting the superiority of ultrasound-based estimation of SSD over traditional anthropometric methods in adult patients undergoing spinal anesthesia. The findings demonstrate that ultrasonography offers significantly greater accuracy, consistency, and clinical utility, aligning with and expanding upon previous research in this field.

The study revealed that ultrasonography-guided SSD (4.5 ± 0.6 cm) closely approximated the actual needle-measured depth (4.6 ± 0.5 cm), with no statistically significant difference (p = 0.12). In contrast, anthropometric estimation (5.1 ± 0.7 cm) systematically overestimated SSD by an average of 0.5 cm (p < 0.001). This overestimation trend is consistent with findings from Tyagi et al., who reported a similar bias in anthropometric models [16]. The MAE and RMSE for ultrasound (0.3 cm and 0.4 cm, respectively) were substantially lower than those for anthropometry (0.7 cm and 0.9 cm), reinforcing ultrasound’s precision.

Bland-Altman analysis further confirmed the reliability of ultrasonography, with tight limits of agreement (-0.6 to +0.4 cm) and minimal bias (-0.1 cm). These results align with Chauhan et al., who reported comparable agreement (±0.5 cm) in obese patients [17]. The strong intraclass correlation coefficient (ICC) of 0.91 and Pearson correlation (r = 0.92) for ultrasonography-guided SSD further validate its clinical applicability, as noted in studies by Kumar et al. [18] and Chauhan et al. [19].

A critical finding was the differential performance of anthropometric and ultrasound-based methods across BMI categories. In normal-weight patients (BMI 18.5-24.9), anthropometric errors were modest (0.5 ± 0.3 cm). However, in overweight (BMI 25-29.9) and obese (BMI ≥30) patients, errors escalated to 0.8 ± 0.4 cm and 1.1 ± 0.5 cm, respectively. This trend mirrors observations by Chauhan et al., who attributed anthropometric inaccuracies in obese patients to abnormal fat distribution [19]. In contrast, ultrasonography maintained consistent accuracy across all BMI groups, with errors never exceeding ±0.5 cm, as corroborated by Girimurugan et al. [20].

The bimodal error distribution in anthropometry, revealed by kernel density plots, underscores its inconsistency, particularly in high-BMI populations. At the 90th percentile, anthropometric errors surpassed 1.3, a clinically significant margin that could lead to procedural failures or complications. These findings align with observations of Broadbent et al., who reported a 29% misidentification rate for lumbar interspaces using palpation-based methods [21]. Ultrasonography mitigates these risks by providing real-time anatomical visualization, reducing reliance on probabilistic formulas.

Despite adding a few seconds for pre-procedural scanning, ultrasound contributed to an overall reduction in procedural time by minimizing needle redirections and improving first-pass success. Similar efficiency gains were reported by Gayathri et al., who noted a 30% reduction in total procedure time with ultrasound use [22]. These benefits are particularly pronounced in high-risk populations, such as obese patients and those with spinal deformities, where landmark-based techniques are notoriously unreliable.

ROC analysis highlighted ultrasonography’s superior discriminative ability for identifying difficult cases (SSD >5 cm), with an AUC of 0.93, sensitivity of 94.1%, and specificity of 88.3%. In contrast, anthropometry performed poorly (AUC = 0.69), limiting its utility in clinical decision-making. These results are consistent with Perlas et al.’s meta-analysis, which affirmed ultrasound’s high diagnostic accuracy in neuraxial block planning [23].

The ability to predict difficult spinal access is particularly valuable in obstetric and trauma settings, where procedural delays can have catastrophic consequences. Ultrasound’s high sensitivity ensures that clinicians can anticipate challenges and adjust their approach accordingly, improving patient safety.

Despite its advantages, ultrasonography is not without limitations. Operator dependence remains a significant barrier, as image acquisition and interpretation require specialized training. Conroy et al.'s study emphasizes this operator dependency [9]. Additionally, variations in transducer type, scanning plane, and measurement endpoints (e.g., posterior vs. anterior dura) can introduce intra-observer variability, as noted by Arzola et al. [10]. Equipment accessibility is another challenge, particularly in resource-limited settings where ultrasound machines may be unavailable. Anthropometric models, despite their limitations, remain a viable alternative in such environments, provided they are population-specific and regularly validated.

Emerging technologies, such as AI-powered SSD prediction models, offer promising solutions to bridge the gap between convenience and accuracy. Future research should focus on standardizing ultrasound protocols to minimize variability in measurements. Expanding training programs to enhance operator proficiency and accessibility. Developing AI-assisted tools for real-time SSD estimation, particularly in low-resource settings. Conducting multicentric studies to validate hybrid models across diverse populations.

Limitations of the study

The present study has certain limitations. The relatively small sample size reduces its statistical power and limits generalizability. Being a single-centre study, the findings may not be applicable to populations with different anthropometric or ethnic characteristics. Ultrasound measurements are operator-dependent, and accuracy may vary with the examiner’s skill and experience. In addition, patient-related factors such as obesity, spinal deformities, and poor bony landmarks can influence both ultrasound and anthropometric estimations. Finally, measurement bias may occur as the actual needle depth can vary with needle angle, tissue compression, and patient positioning.

## Conclusions

This study reinforces the clinical superiority of ultrasound-based SSD estimation over anthropometric methods. Ultrasound’s accuracy, consistency, and ability to predict difficult cases make it an indispensable tool in modern spinal anesthesia. While challenges such as operator dependence and equipment requirements persist, ongoing advancements in AI and standardized training protocols are poised to address these barriers. Anthropometric models, though convenient, should be used judiciously and supplemented with imaging where possible. Future innovations in this field hold the promise of further optimizing spinal anesthesia outcomes, ensuring safer and more efficient patient care.
